# The predictive value of procalcitonin for postoperative early pancreatic fistula

**DOI:** 10.1186/s12893-020-00755-2

**Published:** 2020-05-06

**Authors:** Quanyu Zhou, Yuxiao Xia, Zehua Lei

**Affiliations:** 1 Department of Hepatobiliary and Pancreatic Surgery, The People’s Hospital of Leshan City, Leshan, Sichuan 614000 People’s Republic of China; 2 Department of Nuclear Medicine, The Affiliated Hospital, Southwest Medical University, No 15 TaiPing St, Jiangyang District, Luzhou, 64600 Sichuan People’s Republic of China

**Keywords:** Pancreaticoduodenectomy, Postoperative pancreatic fistula, Procalcitonin

## Abstract

**Background:**

To investigate the early prediction value of procalcitonin (PCT) in pancreatic fistula (POPF) after pancreatoduodenectomy (PD).

**Method:**

Retrospective analysis of clinical data of 67 patients undergoing pancreaticoduodenectomy (PD) and 19 patients undergoing distalpancreatectomy (DP) were performed in the Department of Hepatobiliary Surgery, Leshan People’s Hospital from January 2017 to December 2018. All patients were divided into POPF group and non-POPF group depending on the presence of pancreatic fistula. And fistulas were classified according to the ISGPF classification scheme. Plasma PCT levels, serum CRP concentration, and WBC counts were assessed preoperatively and on postoperative days (PODs) 1, 3, and 5. Statistical analyses were performed with statistical software. The ROC curve was used to analyze the efficacy of PCT and CRP in POPF prediction after surgery and determine their Cut-off value.

**Result:**

There were no statistically significant differences identified in age, gender, BMI, diabetes, abdominal surgery history, preoperative laboratory data, operation time, intraoperative bleeding volume, tumor nature and medical expenses of PD patients between the two groups (*P* > 0.05). While the incidence of postoperative hyperglycemia, postoperative ICU rate and postoperative hospital stay were statistically significant (*P* < 0.05). The AUC for PCT diagnosis of pancreatic fistula 1 day after surgery was 0.77 (95% CI: 0.675 ~ 0.860). Compared with CRP [0.53 (95% CI: 0.420 ~ 0.639)] and WBC [0.60 (95% CI: 0.490 ~ 0.705)], the optimal cut-off value (cut-off) was 0.67 μg/L. At this time, the sensitivity and specificity of detecting pancreatic fistula were 73.68 and 76.12%, respectively. The results at 3 days after surgery were similar to those at 5 days after surgery. And DP patients had similar results as PD patients.

**Conclusion:**

The PCT is valuable for early prediction of pancreatic fistula after Pancreaticoduodenectomy.

## Background

Pancreaticoduodenectomy (PD) is the only curative treatment of malignant diseases of the periampullary region of the pancreas and is also recommended for the treatment of premalignant tumors at a high risk of degeneration. In recent years, the perioperative mortality rate has decreased significantly. However, the postoperative complication rate is still as high as 20% ~ 60% [1]. Based on a review of the literature by the ISGPF, a postoperative pancreatic fistula (POPF) was defined as an abnormal communication between the pancreatic ductal epithelium and another epithelial surface containing pancreas-derived, enzyme-rich fluid. And the incidence is 3% ~ 45% [2]. If not properly treated, POPF may lead to celiac infection. Celiac infection not only cause but also can aggravate POPF if it is difficult to control. In addition, poor control of pancreatic fistula and celiac infection can also cause considerable complications, such as intra-abdominal bleeding, gastric emptying disorders, sepsis and other complications. These complications are important because they can result in longer hospitalization, increased hospitalization costs and increased mortality [3]. Therefore, it is great significance to identify reliable parameters that ideally enable effective risk stratification, based on easily available during hospitalization data, in order to adopt strategies tailored to individual situation.

Procalcitonin (PCT), the 116 amino acids long precursor of calcitonin, is abnormally elevated in sepsis. Procalcitonin is regarded as a good diagnostic marker of sepsis in critically ill patients [4]. However the correlation between the level of PCT and the prognosis of POPF is still unclear. So the results of this work may important for extending the knowledge about PCT and its applications as a clinically useful biomarker of infection severity and provide a reference for clinical practice.

## Methods

### General information

The clinical data from 67 patients who underwent pancreaticoduodenectomy (PD) and 19 patients who underwent distal pancreatectomy (DP) were prospectively included in this study from January 2017 to December 2018 in hepatobiliary surgery of leshan people’s hospital (China) were retrospectively analyzed. All patients were divided into POPF group (grade B pancreatic fistula, grade C pancreatic fistula) and non-POPF group depending on the presence of pancreatic fistula. And fistulas were classified according to the ISGPF classification scheme [2]. Patients meeting the following criteria were excluded in the study: (1) ongoing clinical infections; (2) undergoing emergency surgery; (3) accompanied by rheumatism, rheumatoid and other autoimmune diseases; (4) renal insufficiency (blood creatinine≥133 μmol/L); (5) Hepatic Insufficiency (total bilirubin≥200 μmol/L); (6) younger than 18 year.

General information of patients with PD: 43 male patients and 24 female patients; There were 46 cases of pancreatic head carcinoma and 6 cases of benign pancreatic head lesions. There were 7 cases of cholangiocarcinoma and 2 cases of benign bile duct lesions. There were 4 cases of duodenal carcinoma and 2 cases of benign duodenal lesions. General information of DP patients: 4 male patients and 15 female patients; Benign lesions in 8 cases, malignant lesions in 11 cases.

A clinically relevant POPF is defined as a drain output of any measurable volume of fluid with amylase level greater than 3 times the upper Institutional normal serum amylase level, associated with a clinically relevant development/condition related directly to the POPF [2].

### Surgical techniques

Recorded data included demographic characteristics (age and sex), laboratory test findings (basic biochemistry, complete blood count, hepatorenal function), length of hospital stays and postoperative complications. Pancreatoduodenectomy (PD) was performed using the standard Whipple reconstruction. The scope of the resection includes the gallbladder, distal common bile duct, pancreatic head, all upper duodenum and jejunum, as well as nearby lymph nodes and fatty connective tissue, and then used Child sequential reconstruction. The corresponding size of the pancreatic duct stenting tube (6–8 Fr) was used as an endoluminal stent between the main pancreatic duct and jejunal lumen. Finally, drainage tubes were routinely placed near the anastomoses of the pancreas and bile bowel [5].

Distal pancreatectomy (DP) was performed with the standard procedure. The pancreatic parenchyma was divided using electrocautery and blade. The main pancreatic duct was ligated with nonabsorbable sutures and the transected pancreas was occluded with interlocking interrupted polypropylene suture (Prolene; Ethicon Products, USA). A drainage tube was routinely placed near the broken end of the pancreas [6].

Preoperative antibiotic prophylaxis consisted of a preoperative dose, an intraoperative dose, and three postoperative doses of Ceftizoxime (3 g intravenous).

### Measurement of biomarkers

PCT and CRP were compared between POPF and non-POPF groups to explore respective predictive value for pancreatic fistula. Procalcitonin and C-reactive protein were measured in serum samples that were assessed preoperatively and on postoperative days (PODs) 1, 3, 5. Plasma PCT levels were determined using chemiluminescence assay (Triage meterPro, Berlin, Germany). CRP was determined with a turbidimetric assay (Siemens Healthcare Diagnostics). The detection operations were carried out in strict accordance with the kit instructions.

### Ethics statement

This retrospective study was performed with approval from the Ethics Committee of People’s Hospital of Leshan. A written informed consent from the patients was waived because this study was performed retrospectively.

### Statistical analysis

Statistical analyses were performed with the Graphpad prism 7.0 and Medcalc statistical software. Analysis of continuous variables was presented as mean ± SD ($\overline{x}\pm \mathrm{S}$) and compared by unpaired *t* test. The *X*^2^ test was used to compare the categorical variables. Mann-whitney test was used to compare the non-normal distribution data. The ROC curve was used to analyze the efficacy of PCT and CRP in POPF prediction after surgery and determine their Cut-off value. The area under the curve (AUC) was calculated and AUC > 0.5 was considered as diagnostic significance. All *P* values were two sided, and *P* < 0.05 was considered statistically significant.

## Results

### Patient demographics and postoperative complications

Between January 2017 and December 2018, a total of 86 consecutive patients were enrolled in this study. There were no statistically significant differences identified in age, gender, BMI, diabetes, abdominal surgery history, preoperative laboratory data, operation time, intraoperative bleeding volume, tumor nature and medical expenses of PD patients between the two groups (*P* > 0.05). While the incidence of postoperative hyperglycemia, postoperative ICU rate and postoperative hospital stay were statistically significant (*P* < 0.05). Postoperative complications occurred in 45 PD patients (67.1%, 45/67). Among them, there were 14 cases (20.8%) of POPF, 31 cases (46.2%) of non-POPF, including 6 cases of incision infection, 6 cases of delayed gastric emptying, 5 cases of heart disease, 4 cases of bile fistula, 4 cases of pulmonary infection, 3 cases of adhesive intestinal obstruction, 3 cases of urinary tract infection, and 1 case of postoperative pancreatitis. Fourteen patients with POPF were cured and discharged after conservative treatment such as anti-infection, nutritional support, irrigation and drainage. The non-POPF patients’ complications improved during hospitalization after treatment and they were discharged smoothly.

For DP patients in the two groups, there were no statistically significant differences in age, gender, BMI, diabetes, abdominal surgery history, preoperative laboratory data, operation time, intraoperative blood loss, postoperative hyperglycemia, tumor character, and Hospitalization expenses. While the incidence of postoperative ICU rate and postoperative hospital stay were statistically significant (*P* < 0.05). One or more postoperative complications occurred in 36.8% (7/19) of DP patients. Among them, there were 5 cases of POPF, 1 case of pulmonary infection, 1 case of cardiovascular complications, and 1 case of severe celiac infection and 1 case of Chylous fistula were all cured after active treatment. There were no deaths in this group. The full data of patients’ demographics and characteristics were displayed in Table 1.

**Table 1:** Baseline characteristics of PD and DP patients

Characteristics	PD	POPF groups(*n* = 14)	non-POPF groups(*n* = 53)	*t*	*X*^2^	*P* value	DP	POPF groups(*n* = 5)	non-POPF groups(*n* = 14)	*t*	*X*^2^	*P* value
Age (Year)		58.64 ± 1.74	56.49 ± 1.69	0.62		0.53		53.6 ± 569	49.573.22	0.63		0.53
Gender (male/female)		10/4	33/20		0.40	0.52		1/4	3/11		0.004	0.94
BMI (kg/m^2^)		22.79 ± 0.84	23.25 ± 0.38	0.53		0.59		22.64 ± 0.71	24.65 ± 0.61	1.79		0.09
Diabetes (n)		3	6		0.01	0.90		0	2		0.79	0.37
Previous abdominal surgery		3	16		0.41	0.51		3	5		0.56	0.35
Preoperative bilirubin (μmol/L)		106.32 ± 0.92	166.9 ± 17.61	1.96		0.06		12.99 ± 2.89	8.85 ± 1.43	1.40		0.17
Preoperative albumin(g/L)		37.07 ± 1.79	40.11 ± 0.68	1.87		0.06		39.6 ± 0.74	39.86 ± 0.09	0.56		0.57
Preoperative hemoglobin(g/L)		122.2 ± 4.31	130.3 ± 2.24	1.64		0.10		133.1 ± 3.97	118.3 ± 6.64	1.28		0.21
Operation time (min)		242.5 ± 16.04	237.9 ± 7.98	0.26		0.79		190 ± 20.97	203.7 ± 14.26	0.50		0.62
Intraoperative bleeding (ml)		239.2 ± 25.52	243.5 ± 20.12	0.10		0.91		175.4 ± 28.23	134.9 ± 11.1	1.63		0.12
Postoperative hyperglycemia (n)		6	7		7.27	<0.01		0	3		1.27	0.25
Postoperative hypoproteinemia (n)		5	29		0.00	0.92		0	3		1.27	0.25
Postoperative ICU admission (n)		6	10		6.18	0.01		4	1		10.09	0.001
Tumor character (benign/malignant)		4/10	6/47		2.59	0.10		1/4	7/7		1.36	0.24
Postoperative hospital stay (d)		34.86 ± 3.03	22.08 ± 1.27	4.37		<0.0001		22.8 ± 1.42	16.29 ± 1.08	3.23		0.005
Hospitalization cost (RMB ten thousand)		11.89 ± 1.21	10.94 ± 0.62	0.69		0.48		7.34 ± 0.43	6.91 ± 0.50	0.47		0.64

### Comparison of PCT, CRP, WBC levels during perioperative period

For the PD patients, there was no intergroup difference in PCT, CRP, and WBC between the POPF and non-POPF groups before operation. The PCT level of the POPF group on the l, 3 and 5 days after surgery was significantly higher than that of the non-POPF group, and the difference was statistically significant (*P* < 0.05). The CRP level of the POPF group was significantly higher than that of the non-POPF group on the 3rd and 5th day after surgery, with statistically significant difference (*P* < 0.05). While there was no significant difference in WBC level between the POPF group and the non-POPF group on the l, 3 and 5 days after surgery (*P*>0.05). DP patients had similar changes in PCT, CRP and WBC levels as PD patients. The results are summarized in Table 2.

**Table 2:** Levels (means and standard deviations) of PCT, CRP, and WBC in all patients

PD	Pre-operation	POD1	POD3	POD5	DP	Pre-operation	POD1	POD3	POD5
PCT (μg/L)					PCT (mg/L)				
POPF groups(*n* = 14)	0.20 ± 0.02	1.39 ± 0.29^*^	1.27 ± 0.18^*^	0.66 ± 0.13^*^	POPF groups(*n* = 5)	0.05 ± 0.02	1.19 ± 0.23^*^	1.19 ± 0.23^*^	0.67 ± 0.15^*^
non-POPF groups(*n* = 53)	0.21 ± 0.01	0.55 ± 0.05^*^	0.51 ± 0.05^*^	0.35 ± 0.03^*^	non-POPF groups(*n* = 14)	0.02 ± 0.00	0.29 ± 0.07^*^	0.29 ± 0.07^*^	0.27 ± 0.04^*^
CRP (mg/L)					CRP (mg/L)				
POPF groups(*n* = 14)	12.09 ± 2.57	110.8 ± 15.45	170.1 ± 34.79^*^	102.9 ± 22.07^*^	POPF groups(*n* = 5)	4.73 ± 1.84	116.6 ± 5.96	129.6 ± 16.22^*^	79.11 ± 4.52^*^
non-POPF groups(*n* = 53)	11.23 ± 1.17	109.5 ± 8.04	147.4 ± 11.23^*^	60.24 ± 7.13^*^	non-POPF groups(*n* = 14)	4.35 ± 0.86	110.3 ± 2.72	85.91 ± 10.18^*^	48.21 ± 7.28^*^
WBC (× 10^9^/L)					WBC (×10^9^/L)				
POPF groups(*n* = 14)	7.20 ± 1.20	13.81 ± .2.46	16.16 ± 2.88	15.53 ± 1.77	POPF groups(*n* = 5)	4.41 ± 1.72	7.19 ± 0.93	14.16 ± 3.78	15.32 ± 4.97
non-POPF groups(*n* = 53)	8.00 ± 0.64	17.18 ± 1.50	10.99 ± 1.21	11.43 ± 1.14	non-POPF groups(*n* = 14)	3.57 ± 0.67	8.43 ± 0.85	6.81 ± 0.93	12.58 ± 2.61

*P*<0.05^*^

### (PCT and CRP) ROC analyses for the sensitivity and specificity for the prediction of pancreatic fistula

**Fig. 1:**
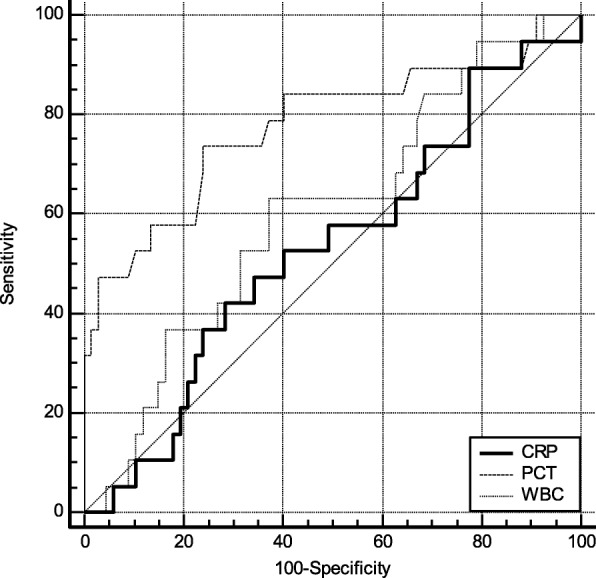
ROC curve for biomarkers in POD 1. PCT has best AUC (0.77), better than CRP and WBC (0.53 and 0.60, respectively)

**Fig. 2:**
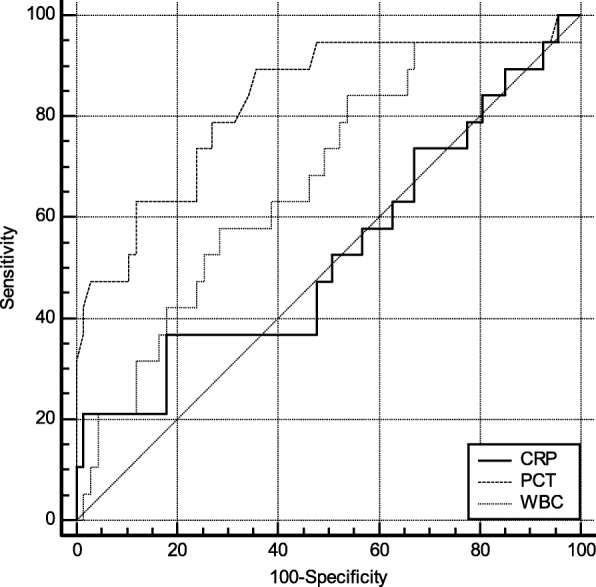
ROC curve for biomarkers in POD 3. PCT has best AUC (0.83), better than CRP and WBC (0.53 and 0.67, respectively)

**Fig. 3:**
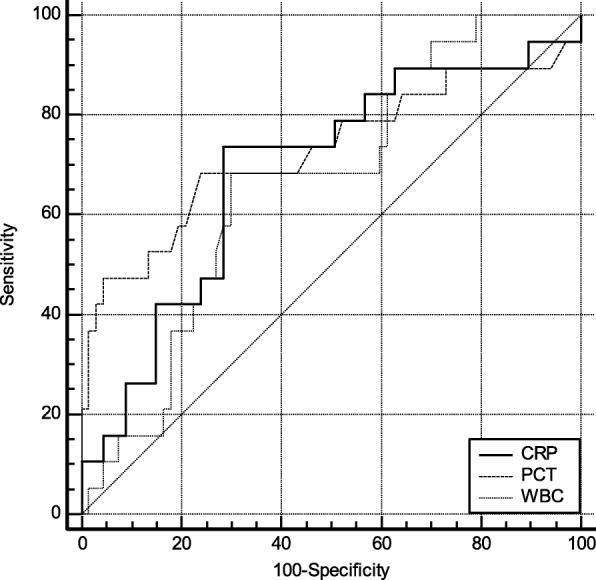
ROC curve for biomarkers in POD 5. PCT has best AUC (0.72), better than CRP and WBC (0.68 and 0.66, respectively)

ROC curve was plotted based on biomarker concentration. According to the results of the ROC curve, the comprehensive degree of sensitivity and specific serum marker detection, and determine the corresponding optimal threshold (Cut-off). As shown in Fig. 1, the AUC for PCT diagnosis of pancreatic fistula 1 day after surgery was 0.77 (95% CI: 0.675 ~ 0.860). Compared with CRP [0.53 (95% CI: 0.420 ~ 0.639)] and WBC [0.60 (95% CI: 0.490 ~ 0.705)], the optimal cut-off value (cut-off) was 0.67 μg/L. At this moment, the sensitivity and specificity of detecting pancreatic fistula were 73.68 and 76.12%, respectively. As shown in Fig. 2, the AUC for PCT diagnosis of pancreatic fistula 3 days after surgery was [0.83 (95% CI: 0.734 ~ 0.902)]. Compared with CRP [0.53 (95% CI: 0.422 ~ 0.641)] and WBC [0.67 (95% CI: 0.563 ~ 0.770)], the optimal cut-off value (cut-off) was 0.56 μg/L. At this moment, the sensitivity and specificity of pancreatic fistula detection were 89.47 and 64.18%, respectively. As shown in Fig. 3, the AUC for PCT diagnosis of pancreatic fistula 5 days after surgery was 0.72 (95%CI: 0.621 ~ 0.818). Compared with CRP [0.68 (95% CI: 0.579 ~ 0.784)] and WBC [0.66 (95% CI: 0.554 ~ 0.762)], the optimal cut-off value (cut-off) was 0.46 μg/L. At this moment, the sensitivity and specificity of detecting pancreatic fistula were 68.42 and 76.12%, respectively. (see Figs. 1, 2, and 3).

## Discussion

Pancreaticoduodenectomy (PD) remains the standard surgical approach for tumors involving the lower bile duct, the pancreatic head, the duodenal papilla, and the ampulla [7]. This operation is considered risky because of high rates of postoperative mortality and complications. Pancreatic fistula after pancreaticoduodenectomy is a common and serious complication and the most important cause of subsequent complications and death after this procedure. The dilemma of pancreatic fistula after pancreaticoduodenectomy has not yet been resolved [7]. Effective control of pancreatic fistula can reduce the occurrence of hemorrhage and celiac infection [8]. The early prevention and identification of high-risk individuals is a crucial strategy in primary prevention of pancreatic fistula after Pancreaticoduodenectomy (PD). At present, there is still a controversy about whether “pancreatic fistula causes infection” or “celiac infection causes or aggravates pancreatic fistula”. Ven FZ et al. believed that pancreatic juice exuded from the broken edge of the pancreas or leaked from the anastomosis of the pancreatic intestine not only can activate bile and intestinal juice and then corroded the surrounding tissues for digestion, but also can complicated with gastrointestinal pathogens entering the abdominal cavity. Therefore, pancreatic fistula can cause and aggravate celiac infection [9]. However, Ng ZQ et al. believed that bacterial translocation can cause uncontrollable celiac infection leading to rapidly progressing pancreatic fistula which can be fatal if not treated in time [10].

CRP is a polypeptide which is an important marker of inflammation, causes precipitation with C-polysaccharide on the cellular wall of pneumococcus and is responsible for activation of classical complement pathway and increased phagocytosis [11]. CRP, often used as an inflammatory and disease activity marker, is a sensitive marker of non-specific inflammatory response [12]. Surgery can lead to a transient increase in PCT, which can gradually decrease to normal within 48 h without co-infection [12]. Ren et al. demonstrated the value of PCT above 0.98 ng/L on POD 3 and 0.83 ng/L on POD 5 could predict the occurrence of IAIs after definitive operations for intestinal fistulae [13]. Giardino et al. showed that CRP and PCT concentration were associated with an increased risk of developing complications and clinical relevant pancreatic fistula after PD. Use of these biomarkers may help identify those patients at highest risk for perioperative morbidity and can provide a good reference for post-PD management [14]. This study retrospectively analyzed the levels of PCT, CRP, and WBC in 67 patients with PD before and 1 day, 3 days, and 5 days after surgery. The AUC for PCT diagnosis of pancreatic fistula 1 day after surgery was 0.77 (95% CI: 0.675 ~ 0.860). Compared with CRP [0.53 (95% CI: 0.420 ~ 0.639)] and WBC [0.60 (95% CI: 0.490 ~ 0.705)], the optimal cut-off value (cut-off) was 0.67 μg/L. At this time, the sensitivity and specificity of detecting pancreatic fistula were 73.68 and 76.12%, respectively. The results at 3 days after surgery were similar to those at 5 days after surgery. There are several advantages of PCT over CRP. The most striking one, demonstrated in this study, is the enormous range of PCT reactivity resulting in a marked increase in PCT plasma levels, especially after 1, 3 days. On the other hand, PCT concentrations are quite low when pancreatic fistula is present. In contrast, CRP levels are often found to be already increased to higher level. Thus, CRP cannot provide information as to further increases in celiac infection and pancreatic fistula, respectively, since it is already increased to its maximum values during a less severe stage of disease.

Further advantages of PCT are its more rapid kinetics; PCT reacts faster than CRP both during an increase or decrease of inflammation. Therefore, PCT can be used as an effective monitoring index for early prediction of pancreatic fistula after PD.

*In addition, in order to effectively eliminate the possibility of PCT elevation during the early postoperative period caused by pancreaticoenteric anastomotic fistula combined with intra-celiac infection caused by enteropathogens. This study introduced 19 patients with distal pancreatectomy (DP). The levels of PCT, CRP and WBC before and after surgery were also analyzed as in the Pancreaticoduodenectomy (PD) group. PCT has also proven its value in early detection of simple pancreatic* “pancreatic fistula with broken pancreatic margin”. Through the comparative analysis of PD and DP patients data. The effect of pancreatic fistula with infection on the specificity value of PCT prediction was indirectly excluded. Therefore, it is further confirmed that PCT is a reliable indicator of POPF’s early prediction. So, we recommend that routine detection of PCT level before and after pancreatic surgery (day 1, 3 and 5), which is conducive to early detection of pancreatic fistula and other related complications. This inference needs to be confirmed by relevant studies in other centers. Meanwhile, according to a previous research, age, gender, BMI, diabetes, pancreatic texture, the diameter of main pancreatic duct, abdominal surgery history, intra-abdominal bleeding, blood transfusion, pancreatojejunostomy, nutritional support, somatostatin, hypoproteinemia and other factors might also be related to the risk factor of POPF after PD [15, 16]. The use of PCT alone as an indicator for predicting pancreatic fistula after PD can identify most high-risk patients at an early stage, but there are still a few cases of pancreatic fistula with no significant change in postoperative PCT levels. Therefore, PCT alone as a predictor has some limitations. We recommends that multi-factor and multi-index joint evaluation for pancreatic fistula after PD to improve the accuracy of early prediction.

## Conclusions

In conclusion, PCT has early predictive value for the occurrence of pancreatic fistula after PD. Give individualized and accurate postoperative management in time, such as changing antibiotics, adjusting the drainage tube, imaging examination, nutritional support and other measures to prevent the occurrence and development of postoperative complications. However, it has the following limitations: (1) This is a retrospective observational study, and sample size is relatively small due to its being conducted in a single center; (2) use of antibiotic drugs by some patients before inclusion in this study may have altered some biomarkers; (3) we measured PCT and CRP values only once. Therefore, we look forward to improving it in the follow-up work.

## Data Availability

The datasets used and/or analysed during the current study are available from the corresponding author on reasonable request (Email:leitseha@126.com).

## Abbreviations

PCT: Procalcitonin
POPF: Pancreatic fistula
PD: Pancreaticoduodenectomy
DP: Distal pancreatectomy
